# Evaluation of 4 Different Suture Threads in A Sample of Dentistry Suture‐Naïve Students: A Randomised Controlled Trial

**DOI:** 10.1111/eje.70134

**Published:** 2026-03-13

**Authors:** Simeone Davide, Marcattili Domenico, Casalena Fabio, Tarallo Francesco, Liguori Margherita Giorgia, Marchetti Enrico

**Affiliations:** ^1^ Department of Life, Health and Environmental Sciences University of L'Aquila L'Aquila Italy

**Keywords:** dental education, preclinical training, randomised controlled trial, student assessment, suture materials

## Abstract

**Introduction:**

Suturing is a crucial step in oral surgery, directly affecting wound healing quality. Simple interrupted suture is widely used in dentistry, often performed by novices using silk sutures due to their ease of use and low cost. However, several alternatives could also be suitable for less experienced practitioners.

**Methods:**

Dental students attending the University of L'Aquila with no prior suturing experience were assigned to use each of the four suture materials on pig hemi‐mandibles in a random order. Silk, Polyamide, Polyglycolic acid and Polytetrafluoroethylene were tested. Subjective feedback was collected through Likert‐scale questionnaires assessing handling, knot security and ease of removal from 101 subjects (study A). Objective performance was evaluated using a structured OSCE‐based assessment, focusing on key suturing phases such as needle handling, suture passage, knot formation and flap approximation in a population of 60 students randomly selected among the considered subjects (study B).

**Results:**

Statistically significant differences (*p* < 0.05) were found in specific aspects as tissue passage resistance, suture manoeuvrability, first knot execution and flap approximation, favouring some materials over silk. Although many other parameters did not reach statistical significance, the data consistently showed that silk did not outperform the alternative materials in any domain, suggesting that its common use is not supported by superior handling or performance.

**Conclusion:**

Silk does not provide superior handling compared to the other tested materials. Universities' training programmes should incorporate alternative suture materials to improve students' technical skills and the overall quality of suturing.

## 1 Introduction

Suturing plays a crucial role in dental and oral surgical procedures, significantly influencing the success of wound healing. The choice of suture material, along with the appropriate suturing technique, is fundamental in ensuring optimal tissue approximation, minimising the risk of infection and promoting efficient healing [1]. Factors as the biological properties of the suture, its tensile strength, absorption rate and biocompatibility must be carefully considered to match the specific clinical scenario. A thorough understanding of these aspects is essential for clinicians to make informed decisions that enhance both functional and aesthetic results, ultimately improving patient care.

Several methods and materials for achieving excellent wound closure have been reported in the literature [2]. The variety of anatomical structures, access to the relevant tissues, specific tongue mobility, restricted access and swallowing patterns can influence the ease of surgery and the quality of wound closure [1]. Beyond these constraints, the edges of the incision must be brought together as close as possible to allow for better wound healing and guarantee that the clot remains stable enough to withstand mechanical forces [3]. The stability of the clot and early tissue healing are in fact essential to avoid dehiscence and promote predictable wound closure [4, 5]. This goal is achieved by keeping the wound edges tightly approximated [6].

The best way to achieve optimal wound closure is through suturing, which stabilises wound edges. Consequently, suturing techniques represent a procedure of primary importance in surgical practice [7].

Different types of suture materials are available in the market, depending on the type of surgery being performed [7]. At the beginning of the 20th century, silk was the most commonly used material [8]. Silk has excellent tensile strength, although bacterial biofilm easily colonises its surface [9]. In modern periodontology, synthetic sutures are preferred [2, 3, 4, 5, 6, 7, 8, 9, 10]. These materials are classified as absorbable or non‐absorbable and can be either monofilament or multifilament [11]. Monofilament is generally preferred due to its lower capillarity, which reduces the risk of bacterial adhesion and subsequent infections [12].

While numerous studies have explored the biological and mechanical characteristics of different suture materials [1, 2], limited attention has been given to how these materials perform in the hands of novice operators, particularly dental students at the early stages of their clinical training. This study addresses this gap by investigating both subjective preferences and objective performance in suture‐naive students using four different suture threads. By combining student‐reported perceptions with structured skill evaluations, the study provides a comprehensive and practical insight into how different materials influence learning and execution.

The primary objective was to test the practicality and manageability of four suture materials in an objective manner, free from cognitive biases or personal preferences that might influence material choice. The secondary aim of this study was to determine which of the proposed materials yielded the best quality sutures when performed by inexperienced hands.

## 2 Materials and Methods

### 2.1 Study Setting

Dental students inexperienced in suturing and unaware of the practical and clinical differences among the materials were recruited for this randomised controlled study by the Department of Periodontology of the University of L'Aquila (Italy) until January 2025. To evaluate the subjective satisfaction of participants with each tested material, questionnaires were formulated asking participants to express their degree of agreement or disagreement with a series of statements aimed at investigating certain characteristics of the suture thread, such as thread sliding, manageability and ease of knot closure.

Evaluation tables were prepared to analyse the quality of performance, examining each phase of placing a suture, from needle handling to knot closure.

To ensure anonymity, randomisation and blinding, opaque and sealed envelopes containing the four materials were prepared. Each suture thread package was associated with a number indicating the order in which to use the four materials and a letter corresponding to a subjective evaluation questionnaire, ensuring that the student was unaware of the material being tested while being able to respond to the corresponding questionnaire. To ensure a random order of sutures with each of the four materials, a block randomisation list was generated. The students found the materials and the questionnaire directly in front of their workstations, along with the necessary tools to be used.

Each workstation was set up with the sutures:

- Silk (SILK, Sweden & Martina S.p.A., Due Carrare, PD, Italy)
- Polyamide (POLINYL, Sweden & Martina S.p.A., Due Carrare, PD, Italy)
- Polyglycolic acid (RESORB, Sweden & Martina S.p.A., Due Carrare, PD, Italy)
- Polytetrafluoroethylene (MONOSOFT, Sweden & Martina S.p.A., Due Carrare, PD, Italy)

To ensure that the evaluations were based solely on the suture material, without other potential factors that could jeopardise the results, all the sutures had the same needle in terms of cross‐section, length and shape (5–0, 13 mm, 3/8 circle, triangular needle).

A hemi‐mandible of pig, and a kit of surgical instruments consisting of straight round scalpel handle 15c blade, Castroviejo straight needle holder, Prichard periosteal elevator, straight tissue pliers and surgical scissors (Hu‐Friedy Italy S.R.L.) were also disposed.

Given the absence of prior training in oral surgical procedures and suturing, participants attended a live demonstration performed by an experienced examiner (D.M.) before the assessment. In addition, a standardised instructional video demonstrating the fundamental steps of a simple interrupted suture was continuously displayed in loop for the full duration of the practical session.

101 students were asked to suture the flaps with four simple interrupted stitches, one with each test material, and to complete the corresponding questionnaires (study A). Data from the evaluation sheets and student feedback were collected. At the same time, random students among them were observed and evaluated by 4 expert tutors, not involved in the study, for each suture performed (study B).

### 2.2 Data Collection

The trial was based on a famous approach widely adopted in medical schools known as Objective Structured Clinical Examination (OSCE), an assessment method consisting of a series of tests called ‘stations’ that the student must complete in succession [13]. In each station, students were required to perform a predefined task corresponding to a specific clinical competence. To evaluate the performance of each test, the optimal expected performance was predetermined, and evaluation tools were constructed based on that execution to ensure objectivity. Thus, to examine the practical skill in performing a suture, an evaluation table was prepared to consistently examine each phase of the manoeuvre, with the aim of objectively defining the level of proficiency in suturing technique (Figure 1).

**FIGURE 1:**
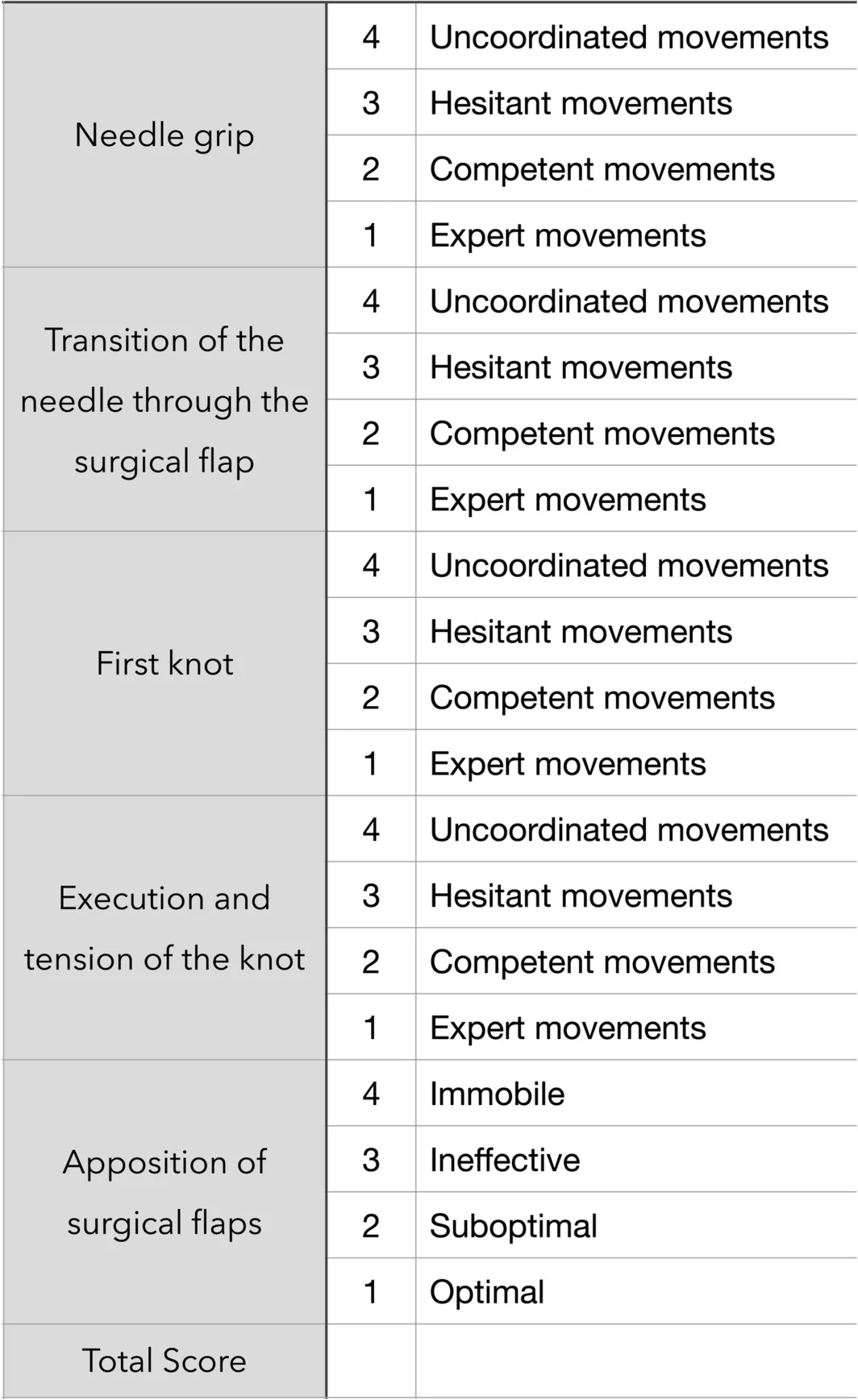
Evaluation table for supervisors' evaluation.

Each station was assigned a score from 1 to 4, examining execution and movement ergonomics, where 1 was the best score achievable while 4 was the worst. Each score was accompanied by a brief explanation to help and guide the examiners towards a more homogeneous evaluation.

In addition to the quality of the sutures performed, favour or disfavour regarding the material's manageability for each student was evaluated. The Likert scale, an instrument developed by American psychometrician Rensis Likert in 1932 for measuring opinions and attitudes, was used for the purpose [14]. This evaluative metric consisted of a series of statements semantically related to the attitudes being investigated. The respondents were asked to express their degree of agreement with each statement, indicating among five multiple choices whether they were: completely in agreement, in agreement, uncertain, in disagreement, or completely in disagreement. Each response was assigned a numerical score from 1 (completely in agreement) to 5 (completely in disagreement) so that the sum of the scores (fifty points maximum) provided the examiners with a clear idea of the individual's stance on the investigated concept. For this purpose, 10 statements (Figure 2) were prepared to investigate the student's subjective perception of the material's manageability, inviting them to evaluate the thread's sliding, tissue trauma, material memory, knot closure ease, knot security and suture removal manoeuvre. The questionnaires were administered to the students via Google Forms, a website to create online forms and surveys with multiple question types and analyse results in real‐time from any device.

**FIGURE 2:**
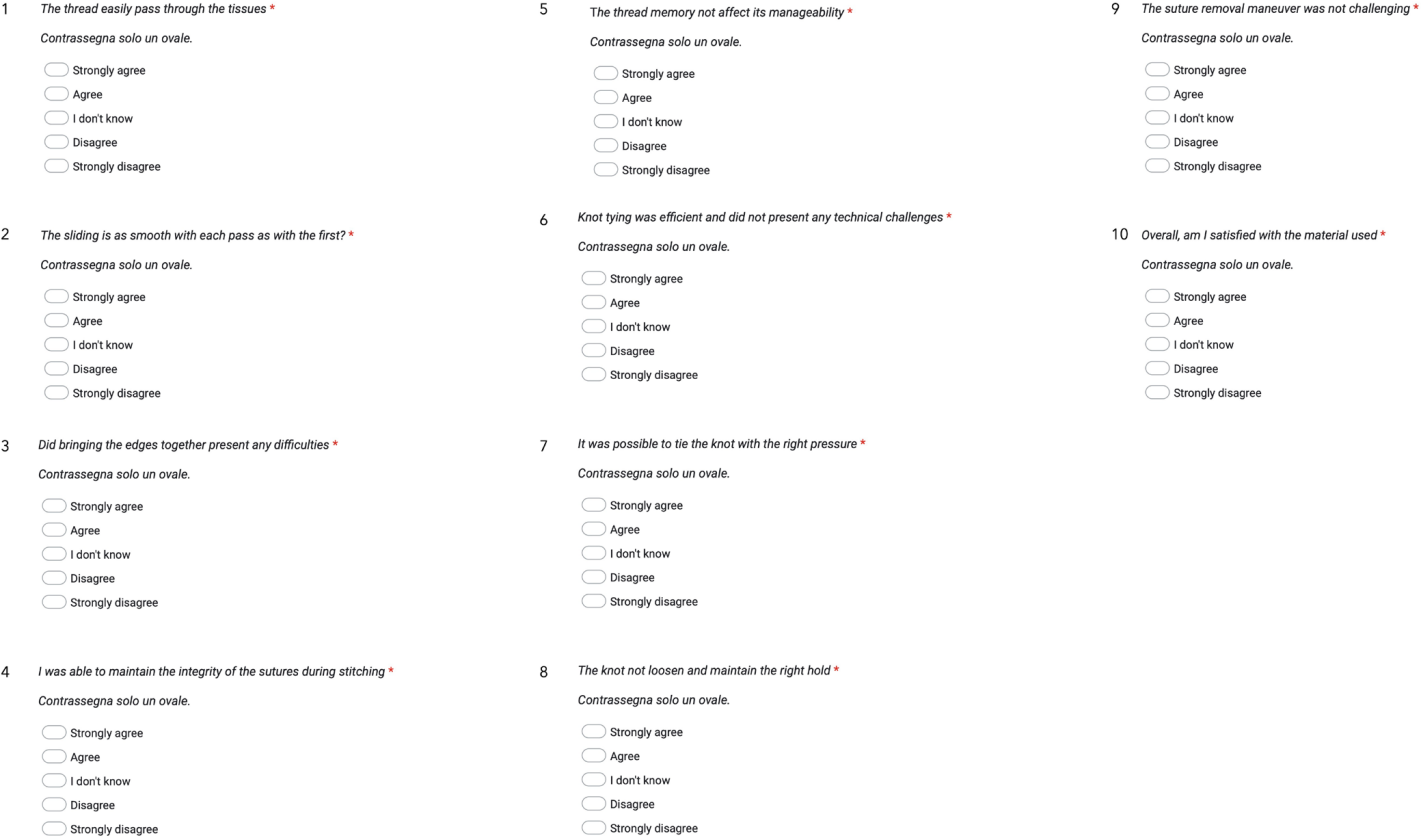
Likert scale for students' evaluation.

### 2.3 Statistical Analysis

Descriptive statistics were computed for all variables: absolute and relative frequencies for ordinal variables, and mean, standard deviation, median, interquartile range and range for continuous data.

For the students' evaluations, responses to 10 Likert‐scale questions regarding usability of suturing materials were compared across four materials using the Kruskal–Wallis test. Where significant differences were identified, pairwise comparisons were conducted using the Mann–Whitney U test with Bonferroni correction for multiple comparisons.

For the supervisors' evaluations, each student was assessed on five suturing skills using a 4‐point Likert scale. An overall score was calculated as the average of the five skill ratings. Skill scores across the four materials were compared using the Friedman test, followed by Wilcoxon signed‐rank tests with Bonferroni correction for post hoc pairwise comparisons.

The significance level was set at α = 0.05. A post hoc power analysis was performed to evaluate the statistical power of the primary student evaluation sample to detect a medium‐to‐large effect size (*f* = 0.33) with 95% confidence.

## 3 Results

### 3.1 Participants

The whole population fulfilled the tasks. A total of 101 students completed the questionnaire assessing the usability of four different suture materials (Study A), while 60 students were evaluated by supervisors on five core suturing skills (Study B). Results are presented separately for each study, beginning with the analysis of student‐reported outcomes and followed by the supervisor‐based assessment of technical performance.

### 3.2 Study ‘a'

QUESTION 1 (‘Does the thread easily pass through the tissues?’) (Graph 1, Table 1).

**Graph 1:**
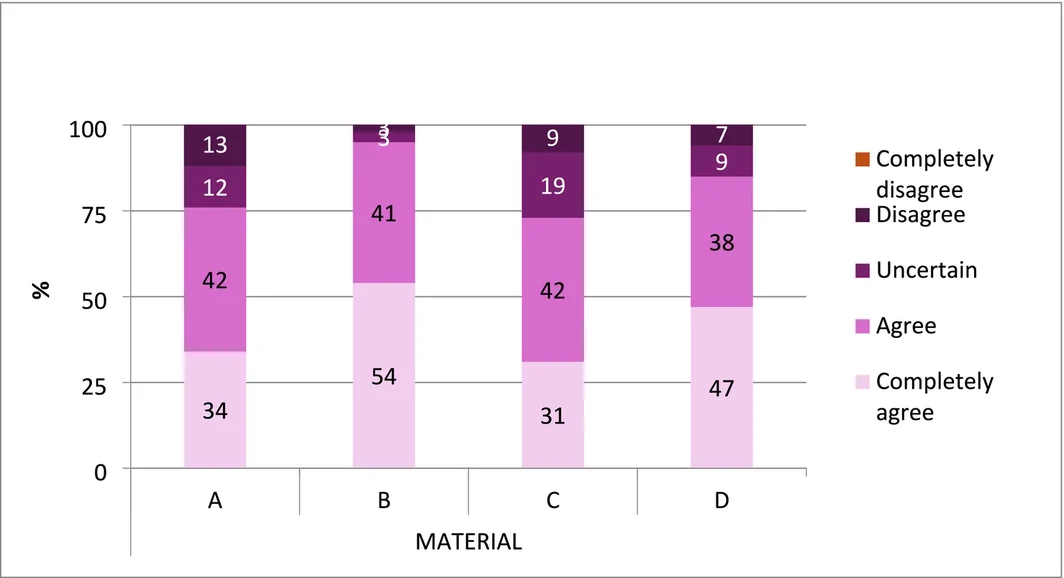
Ease of Tissue Passage (Student Evaluation—Question 1). Distribution of students' responses to the question ‘Does the thread easily pass through the tissues?’

**TABLE 1:** Pairwise Comparisons for Tissue Passage (Question 1).

	A	B	C	D
A				
B	< 0.001***			
C	1.000	< 0.001***		
D	0.216	0.782	0.072	

*Note:* Statistical comparison of students' responses regarding ease of tissue passage. Polyamide (B) showed significantly higher agreement than silk (A) and polyglycolic acid (C) (A vs. B: *p* < 0.001, B vs. C: *p* < 0.001). No other pairwise comparisons were significant. A. Silk; B. Polyamide; C. Polyglycolic acid; D. Polytetrafluoroethylene.

The Overall analysis among the four threads showed a statistically significant difference (*p* < 0.001). In pairwise analyses, thread A (SILK) versus thread B (A vs. B, *p* < 0.001) and thread B versus thread C (B vs. C, *p* < 0.001) are statistically different. Substantially, the level of agreement with material B is significantly higher than with materials A and C (*p* < 0.001). All other combinations are not statistically significant (A vs. C, *p* = 1.000; A vs. D, *p* = 0.216; B vs. D, *p* = 0.782; C vs. D, *p* = 0.072).

QUESTION 2 (‘Is the sliding as smooth with each pass as with the first?’).

The Overall analysis among the threads did not show a statistically significant difference (*p* = 0.062). No statistically significant differences were found in pairwise analyses either (A vs. B, *p* = 0.102; A vs. C, *p* = 1.000; A vs. D, *p* = 0.516; B vs. C, *p* = 0.330; B vs. D, *p* = 1.000; C vs. D, *p* = 1.000).

QUESTION 3 (‘Did bringing the edges together present any difficulties?’).

The Overall analysis among the four threads did not show a statistically significant difference (*p* = 0.857). No statistically significant differences were found in pairwise analyses either (A vs. B, *p* = 1.000; A vs. C, *p* = 1.000; A vs. D, *p* = 1.000; B vs. C, *p* = 1.000; B vs. D, *p* = 1.000; C vs. D, *p* = 1.000).

QUESTION 4 (‘Was I able to maintain the integrity of the sutures during stitching?’).

No statistically significant difference was found among the threads (*p* = 0.479). No statistically significant differences were found in pairwise analyses either (A vs. B, *p* = 1.000; A vs. C, *p* = 1.000; A vs. D, *p* = 1.000; B vs. C, *p* = 1.000; B vs. D, *p* = 1.000; C vs. D, *p* = 1.000).

QUESTION 5 (‘The thread memory not affect its manageability’) (Graph 2, Table 2).

**Graph 2:**
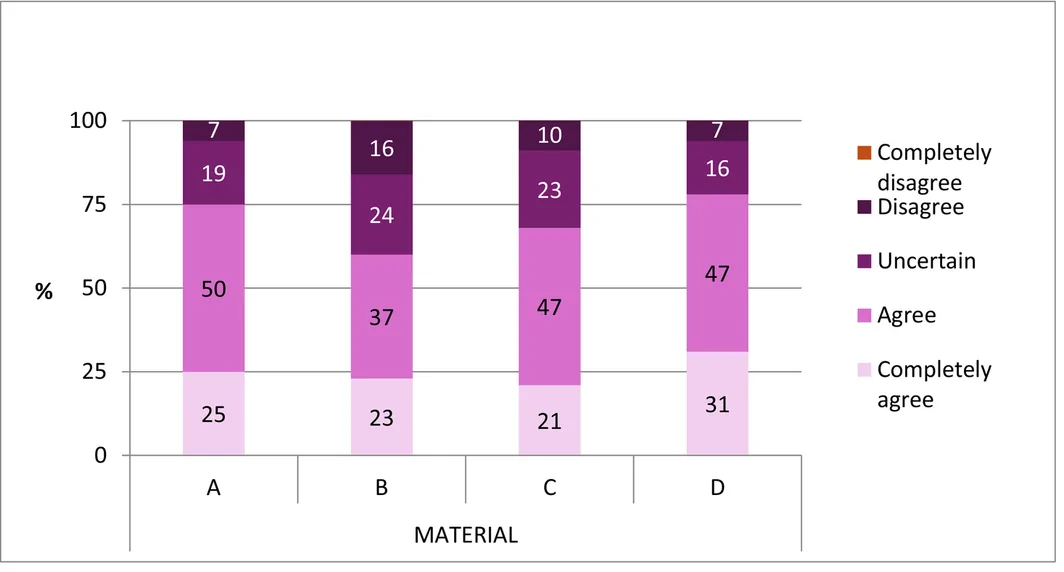
Impact of Thread Memory on Handling (Student Evaluation—Question 5). Distribution of students' responses to the question ‘Thread memory did not affect its manageability’.

**TABLE 2:** Pairwise Comparisons for Thread Memory (Question 5).

	A	B	C	D
A				
B	0.390			
C	1.000	1.000		
D	1.000	0.066	0.342	

*Note:* Pairwise comparisons of student ratings on the impact of thread memory. Although overall significance was reached (*p* = 0.044), no statistically significant pairwise differences were found. The closest to significance was B vs. D (*p* = 0.066). A. Silk; B. Polyamide; C. Polyglycolic acid; D. Polytetrafluoroethylene.

The threads showed a statistically significant difference in the Overall analysis (*p* = 0.044). No statistically significant differences were found in pairwise analyses either (A vs. B, *p* = 0.390; A vs. C, *p* = 1.000; A vs. D, *p* = 1.000; B vs. C, *p* = 1.000; B vs. D, *p* = 0.066; C vs. D, *p* = 0.342).

QUESTION 6 (‘Knot tying was efficient and did not present any technical challenges’) (Graph 3, Table 3).

**Graph 3:**
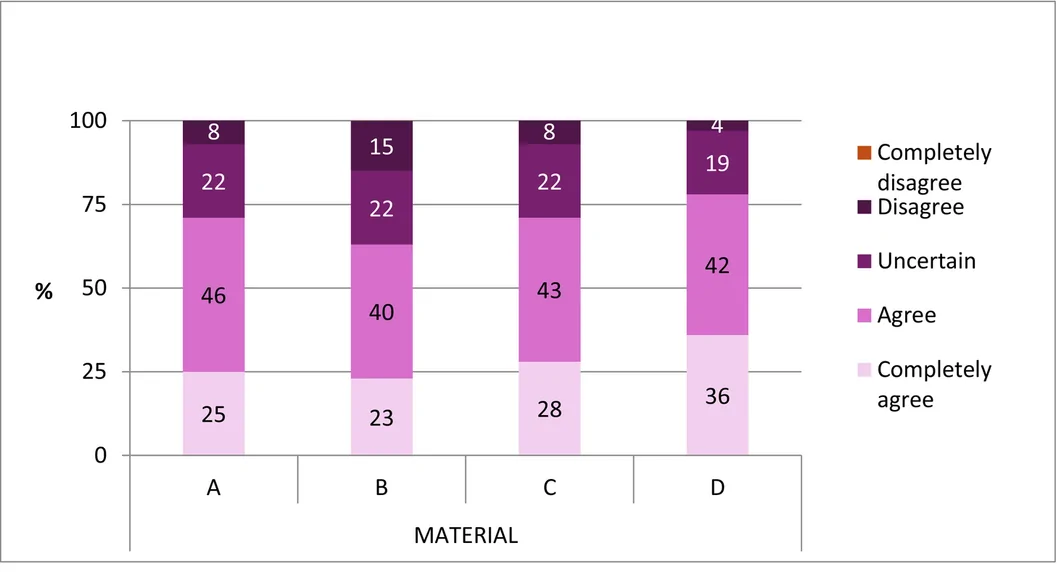
Knot Tying Efficiency (Student Evaluation—Question 6). Distribution of students' responses to the question ‘Knot tying was efficient and did not present any technical challenges’.

**TABLE 3:** Pairwise Comparisons for Knot Tying Ease (Question 6).

	A	B	C	D
A				
B	1.000			
C	1.000	1.000		
D	0.462	0.030*	0.864	

*Note:* Comparative analysis of student feedback on knot‐tying difficulty. PTFE (D) was rated significantly better than polyamide (B) (*p* = 0.030). No other pairwise differences reached statistical significance. A. Silk; B. Polyamide; C. Polyglycolic acid; D. Polytetrafluoroethylene.

The Overall analysis among the 4 threads showed a statistically significant difference (*p* < 0.039). In pairwise analyses, the only statistically significant data was between thread B and thread D (*p* = 0.030). The other 5 combinations did not show statistically significant data (A vs. B, *p* = 1.000; A vs. C, *p* = 1.000; A vs. D, *p* = 0.462; B vs. C, *p* = 1.000; C vs. D, *p* = 0.864).

QUESTION 7 (‘Was it possible to tie the knot with the right pressure?’).

The Overall analysis among the 4 threads did not show a statistically significant difference (*p* = 0.242). No statistically significant differences were found in pairwise analyses either (A vs. B, *p* = 1.000; A vs. C, *p* = 1.000; A vs. D, *p* = 0.480; B vs. C, *p* = 1.000; B vs. D, *p* = 0.822; C vs. D, *p* = 0.498).

QUESTION 8 (‘Does the knot not loosen and maintain the right hold?’).

The Overall analysis among the 4 threads did not show a statistically significant difference (*p* = 0.242). No statistically significant differences were found in pairwise analyses (A vs. B, *p* = 1.000; A vs. C, *p* = 1.000; A vs. D, *p* = 0.552; B vs. C, *p* = 1.000; B vs. D, *p* = 1.000; C vs. D, *p* = 1.000).

QUESTION 9 (‘Was the suture removal maneuver without difficulties?’).

The Overall analysis among the 4 threads did not show a statistically significant difference (*p* < 0.039). No statistically significant differences were found in pairwise analyses either (A vs. B, *p* = 1.000; A vs. C, *p* = 1.000; A vs. D, *p* = 1.000; B vs. C, *p* = 1.000; B vs. D, *p* = 1.000; C vs. D, *p* = 1.000).

QUESTION 10 (‘Overall, am I satisfied with the material used?’).

In the last question, no statistically significant differences were found among the 4 threads (*p* = 0.688). No differences were found in pairwise analyses either (A vs. B, *p* = 1.000; A vs. C, *p* = 1.000; A vs. D, *p* = 1.000; B vs. C, *p* = 1.000; B vs. D, *p* = 1.000; C vs. D, *p* = 1.000).

### 3.3 Study ‘b'

SKILL 1 (‘Needle grip’): No statistically significant differences were found either among the 4 threads (*p* = 0.464). In the pairwise analyses among the various threads, the result was the same (A vs. B, *p* = 1.000; A vs. C, *p* = 1.000; A vs. D, *p* = 1.000; B vs. C, *p* = 1.000; B vs. D, *p* = 1.000; C vs. D, *p* = 1.000).

SKILL 2 (‘Transition of the needle through the surgical flap’): No statistically significant differences were found either among the 4 threads (*p* = 0.118) or in pairwise analyses (A vs. B, *p* = 1.000; A vs. C, *p* = 1.000; A vs. D, *p* = 1.000; B vs. C, *p* = 0.054; B vs. D, *p* = 0.732; C vs. D, *p* = 1.000).

SKILL 3 (‘First knot’) (Graph 4, Table 4): The Overall analysis showed a statistically significant difference (*p* = 0.005). In the pairwise analyses of skill 3, the only statistically significant comparison was found between thread B and thread D (*p* = 0.006). The other pairwise comparisons were not statistically significant (A vs. B, *p* = 0.060; A vs. C, *p* = 1.000; A vs. D, p = 1.000; B vs. C, *p* = 0.738; C vs. D, *p* = 0.240).

**Graph 4:**
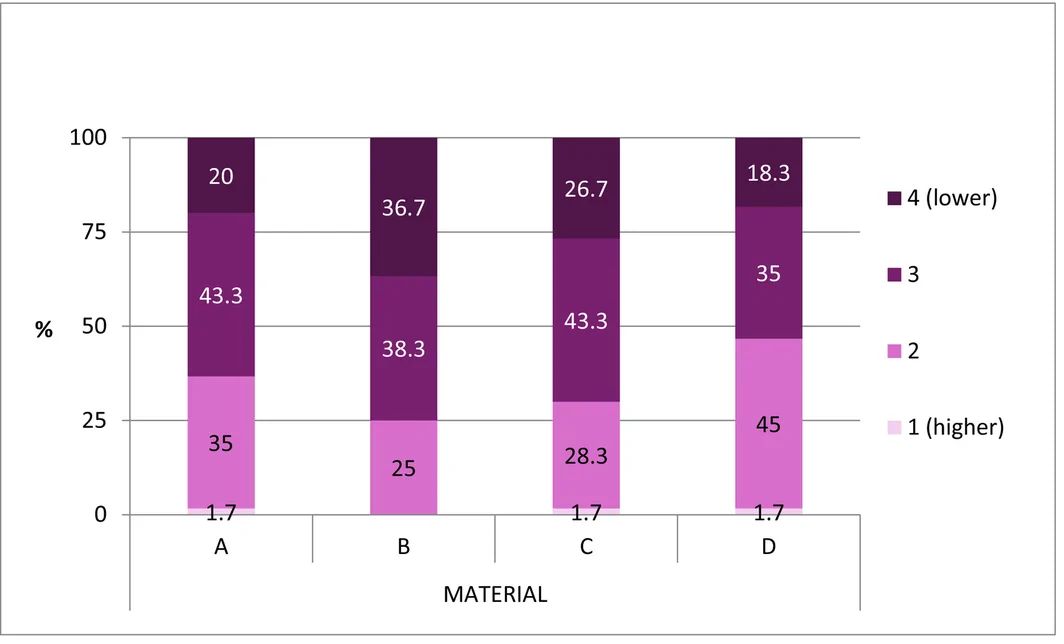
First Knot Quality (Supervisor Evaluation—Skill 3). Distribution of supervisor's evaluation to the skill ‘First knot’.

**TABLE 4:** Supervisor Scores for First Knot (Skill 3).

	A	B	C	D
A				
B	0.060			
C	1.000	0.738		
D	1.000	0.006**	0.240	

*Note:* A significant difference was found between polyamide (B) and PTFE (D) (*p* = 0.006), favouring PTFE. A. Silk; B. Polyamide; C. Polyglycolic acid; D. Polytetrafluoroethylene.

SKILL 4 (‘Execution and tension of the knot’): The 4 statistical distributions of the respective threads were marginally heterogeneous (*p* = 0.077). In pairwise analyses, no statistically significant differences were observed (A vs. B, *p* = 1.000; A vs. C, *p* = 1.000; A vs. D, *p* = 0.198; B vs. C, *p* = 0.054; B vs. D, *p* = 0.780; C vs. D, *p* = 1.000).

SKILL 5 (‘Apposition of surgical flaps’) (Graph 5, Table 5): Statistically significant differences were observed among the 4 threads (*p* = 0.026). In pairwise analyses, a statistically significant difference between thread A and thread D (*p* = 0.012) was found. In the remaining pairwise combinations, no statistically significant differences were observed (A vs. B, *p* = 1.000; A vs. C, *p* = 0.846; B vs. C, p = 1.000; B vs. D, *p* = 0.252; C vs. D, *p* = 0.684).

**Graph 5:**
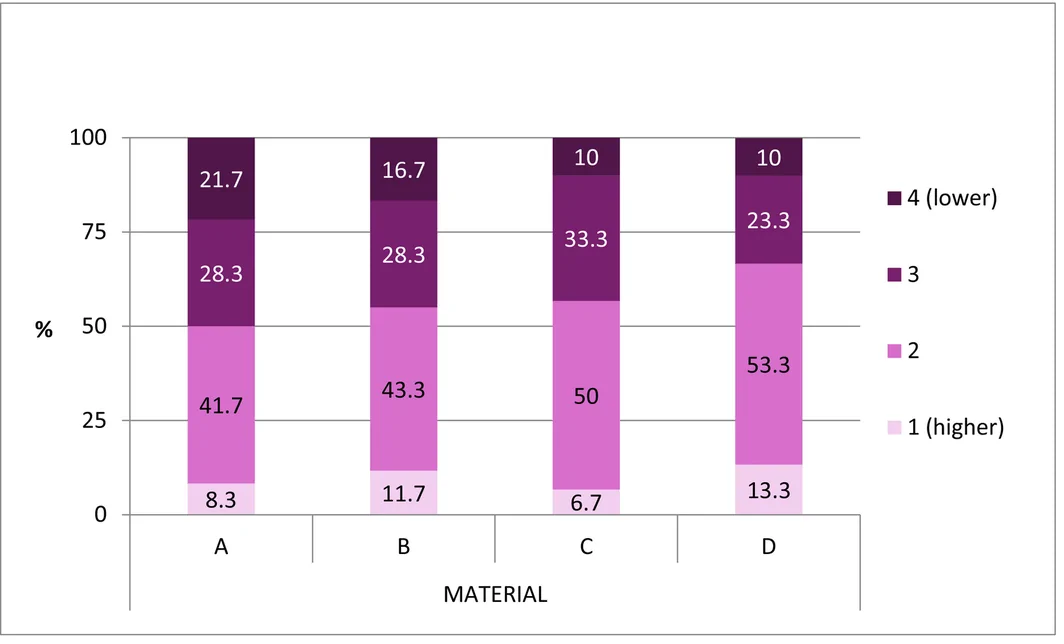
Flap Approximation (Supervisor Evaluation—Skill 5). Scores assigned by supervisors for surgical flap approximation.

**TABLE 5:** Supervisor Scores for Flap Approximation (Skill 5).

	A	B	C	D
A				
B	1.000			
C	0.846	1.000		
D	0.012*	0.252	0.684	

*Note:* PTFE (D) significantly outperformed silk (A) (*p* = 0.012). No other pairwise comparisons were statistically significant. A. Silk; B. Polyamide; C. Polyglycolic acid; D. Polytetrafluoroethylene.

The average score of the 5 skills for thread A was 2.82, for thread B 2.86, for thread C 2.75 and for thread D 2.63 (Graph 6, Table 6).

**Graph 6:**
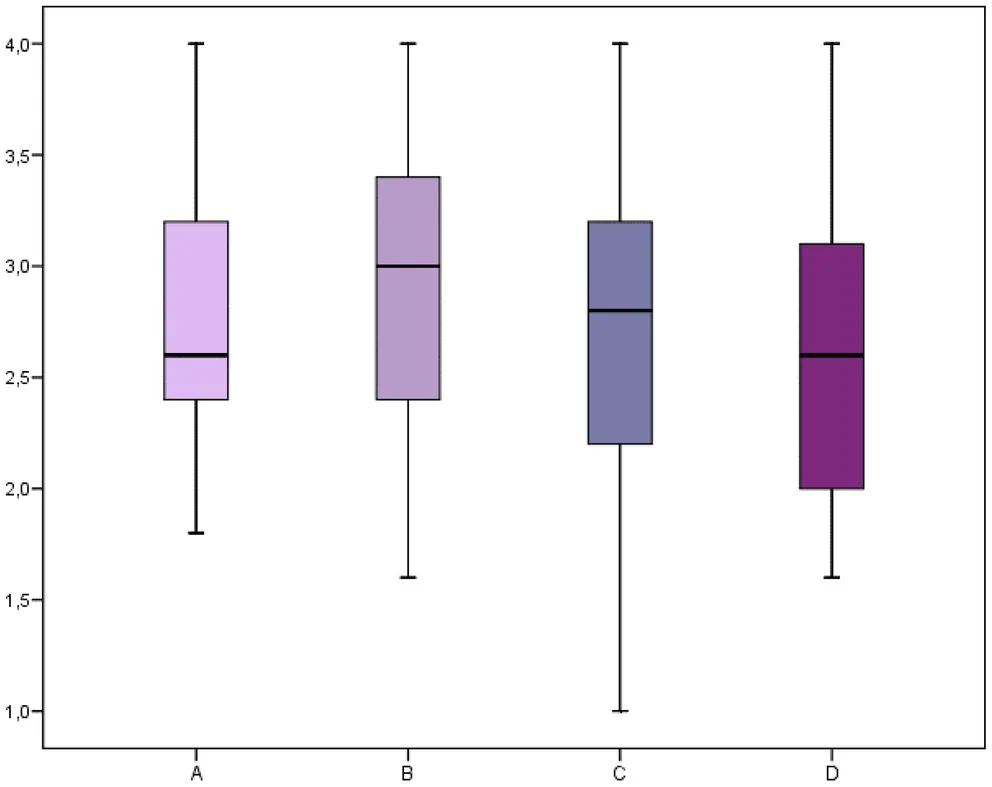
Overall Suturing Skill Score (Supervisor Evaluation). Boxplots showing the overall suturing performance score across all five skills, as rated by supervisors. PTFE (D) achieved the best overall score, followed by polyglycolic acid (C), silk (A) and polyamide (B).

**TABLE 6:** Descriptive Statistics for Overall Skill Score.

	*N*	Mean	Standard deviation	Minimum	Maximum	Percentile 25	Median	Percentile 75
Mean Score A	60	2,82	60	1,80	4,00	2,40	2,60	3,20
Mean Score B	60	2,86	66	1,60	4,00	2,40	3,00	3,40
Mean Score C	60	2,75	66	1,00	4,00	2,20	2,80	3,20
Mean Score D	60	2,63	63	1,60	4,00	2,00	2,60	3,10

*Note:* Mean, standard deviation and distribution percentiles of the overall suturing performance score (average of 5 skills) for each material. PTFE (D) had the lowest (best) mean score (2.63), followed by PGA (C), silk (A) and polyamide (B). A. Silk; B. Polyamide; C. Polyglycolic acid; D. Polytetrafluoroethylene.

## 4 Discussion

The present randomised controlled trial revealed that suture materials commonly perceived as easier to handle, such as silk, did not outperform alternative threads in either subjective or objective evaluations. Notably, polyamide and PTFE demonstrated superior performance in specific aspects of usability and technical execution, suggesting that material properties significantly influence novice suturing outcomes.

Simple interrupted sutures are the most commonly used type of suture for intra‐oral wound closure and arrest of haemorrhage from dental extraction sockets, but other techniques may also be used when appropriate [15, 16]. However, simple interrupted sutures are the first suturing method that dental students encounter when learning how to suture. This approach offers greater flexibility, as an incorrectly tied knot can be removed and redone without compromising the entire suture, thereby allowing for the correction of mistakes made during the learning process. Furthermore, since each knot is independent, a potential failure in one knot does not affect the others, unlike continuous sutures.

In this regard, the primary objective of this study was to compare the manageability of four different types of surgical sutures used by inexperienced dentistry students. Moreover, the authors wanted to compare the students' feedback with the objective evaluation performed by experienced surgical supervisors in order to determine the prevalence of one material over the others when handled by novice operators.

It is well‐known from the literature, in fact, that often the first approach to suture threads by operators is with silk sutures [8]. Silk, therefore, is the most common material in dental surgery thanks to its excellent manageability, knot strength and flexibility but it also has negative aspects [11, 17]. Irritability of healing tissues, accumulation of germs and poor elasticity are the most peculiar aspects [8, 18].

Although it is the most clinically used material, its properties do not make it the ideal and most suitable material for every clinical situation. According to our results, the silk suture resulted comparable to the other materials in all the questions submitted to the students except for question no. 1 where polyamide proved to be statistically significant compared to silk and polyglycolic acid. As a matter of fact, the students found greater resistance in the passage of the silk thread through the tissues, but this characteristic may be attributed to the physical properties of the structure of the silk as multifilament. Moreover, the results of study B demonstrated not statistically significant for all the considered skills except for Skill 5 where the score of silk resulted statistically lower than PTFE. The result may be attributed to the physical properties of the silk as polyfilament which suffered from more friction making it difficult to stick the flaps together with respect to a monofilament thread such as PTFE.

Unlike the previous one, polyamide is a monofilament suture thread. It has the advantages of excellent smoothness and low tissue trauma thanks to the uniformity of its calibre. This type of thread performed exceptionally well, receiving positive feedback from both students and experienced practitioners. The results indicated that polyamide glides smoothly through the tissue, ranking first and being regarded as the thread with the best glide performance. The findings were statistically significant when compared to silk and polyglycolic acid. On the other hand, polyamide exhibited greater thread memory than PTFE, which tended to negatively affect its handling. Although this result was noteworthy, no statistical significance was found. Moreover, the students' responses regarding the ease of knotting the thread revealed that polyamide performed statistically significantly worse than PTFE, probably due to its high elastic memory.

Polyglycolic acid is a multifilament thread coated with a hydrophobic substance. Its braided structure has the advantages of promoting sliding and reducing capillarity but at the same time facilitates the untying of the knot [19]. Furthermore, since it is an absorbable thread, it loses its knot tension and fully reabsorbs in about 60 days [19].

Obviously, since the present trial may be considered an in vitro study, it was not possible to evaluate its efficacy as an absorbable thread over time and during wound healing. In such a scenario, it might have achieved even better results compared to the other threads, which are all non‐absorbable. Regarding the parameters analysed in this study, PGA demonstrated a moderate level of satisfaction among students and was also positively evaluated by experienced practitioners in the assessment of skills. Indeed, no significant differences were found for any pair of materials, although the score of PGA was only marginally higher than polyamide (*p* = 0.054).

Finally, the polytetrafluoroethylene (PTFE) is a synthetic non‐absorbable monofilament that exhibits good smoothness, excellent knot security, and minimal plaque and bacterial accumulation due to its structure. The main drawback is its higher cost that limits its use solely in specific surgical cases. Thanks to its advantages, it is the suture material that was most favoured by students, even though no statistically significant difference was observed. Indeed, when it came to evaluate the smoothness through the tissues, the level of agreement with PTFE was substantially higher than with polyglycolic acid (*p* = 0.072), due to its ability to glide smoothly through the flaps. This capability is attributed to its chemical composition, which makes it slippery, with a low coefficient of friction and non‐stick properties. Moreover, the excellent characteristics of the suture were also confirmed by the sentence regarding the memory affecting the manageability. Although no statistically significant differences were found for any pair of materials, the comparison between polyamide and PTFE showed the strongest difference (*p* = 0.066), as the level of agreement with PTFE was significantly higher than with polyamide. The higher grade of acceptance with PTFE compared to polyamide was also observed about the easiness of the knot tying. In this case, the difference was even statistically significant, demonstrating that PTFE had significantly greater manageability and ease of knot completion compared to polyamide (*p* = 0.030). On the other hand, regarding the skills observed by expert operators, the flaps approximation showed statistically significant relevance (*p* = 0.012). According to the results, PTFE proved to be superior to silk in approximating tissue flaps. This observation is also consistent with the characteristics of the two materials, as PTFE is a monofilament, while silk is a braided multifilament, requiring more effort to bring the wound edges together.

The study is affected by the following limitations: the absence of anatomical structures such as the tongue, cheeks, and reduced buccal opening typical of a real patient could affect the results. The students performed only simple interrupted sutures, which limits the generalizability of the findings. It is plausible that more complex techniques, such as continuous sutures or advanced knot configurations, might lead to different material preferences or performance outcomes. On the other hand, a key strength of the study lies in the recruitment of students with no prior suturing experience, which minimises potential biases linked to preconceptions or established manual habits and ensures a more neutral and unbiased assessment of each suture material.

## 5 Conclusion

Silk is often used in dentistry due to its relatively low cost and the common belief that it is easier to handle. However, our study demonstrates that silk does not offer superior usability or handling compared to the other tested materials. These findings suggest that the choice of suture material should not be based solely on cost but should also take into account clinical and practical considerations to minimise inflammation and optimise the postoperative healing process.

Moreover, training should incorporate alternative suture materials to provide students with a broader understanding of clinical options and improve their technical skills and the overall quality of suturing with biologically superior materials.

## Funding

The authors have nothing to report.

## Conflicts of Interest

The authors declare no conflicts of interest.

## Data Availability

The data that support the findings of this study are available from the corresponding author upon reasonable request.
